# Sliding balloon-assisted thrombectomy combined with aspiration and intrasinus urokinase thrombolysis for the treatment of hemorrhagic cerebral venous sinus thrombosis: experience of 10 patients

**DOI:** 10.3389/fneur.2025.1519308

**Published:** 2025-03-07

**Authors:** Mingsi Zhang, Feixin Jiang, Qingyan Wen, Yiman Chen, Zhiquan Zhang, Min Zhang, Jianxin Zhong

**Affiliations:** ^1^Department of Neurology, Jiangmen Central Hospital, Jiangmen, China; ^2^Department of Neurology, Shenshan Medical Center, Memorial Hospital of Sun Yat-sen University, Shanwei, China

**Keywords:** cerebral venous sinus thrombosis, intracranial hemorrhage, balloon-assisted thrombectomy, aspiration, intrasinus thrombolysis

## Abstract

**Background:**

Cerebral venous sinus thrombosis (CVST) is an unusual cause of stroke. Currently, standard anticoagulant therapy does not have satisfactory efficacy for treating some cases of hemorrhagic CVST. Therefore, we explored the efficacy and safety of the combination of sliding balloon-assisted thrombectomy with aspiration and intrasinus urokinase thrombolysis for the treatment of CVST patients with intracranial hemorrhage (ICH).

**Methods:**

We retrospectively analyzed the clinical, imaging and follow-up data of 10 CVST patients with ICH who underwent sliding balloon-assisted thrombectomy combined with aspiration and intrasinus thrombolysis with urokinase from February 2022 to June 2023. Complete recanalization and partial recanalization in the cerebral venous sinus were defined as imaging outcomes, and the modified Rankin score (mRS) at the 3-month and 6-month follow-ups was used to evaluate clinical efficacy.

**Results:**

A total of 10 CVST patients aged 18–68 years were enrolled, including 5 males. All of the patients was diagnosed with ICH by noncontrast CT and with thrombosis at 3 or more venous sinuses by digital subtraction angiography (DSA). After treatment, complete recanalization was achieved in 6 patients, and partial recanalization was achieved in 4 patients. At the 3- and 6-month follow-up, all 10 patients showed neurological independence (mRS score ≤ 2), without any signs of symptom aggravation, cerebral hematoma enlargement, pulmonary embolism or other complications after treatment.

**Conclusion:**

These results indicated that the combination of sliding balloon-assisted thrombectomy, aspiration and intrasinus urokinase thrombolysis may be safe and effective for the treatment of CVST patients with intracranial hemorrhage.

## Introduction

Cerebral venous sinus thrombosis (CVST) is an unusual cerebrovascular disorder that includes dural sinus thrombosis which refers to clots in large, dural venous conduits and cortical vein thrombosis, accounting for 0.5–3% of strokes (1). Recent population-based studies have shown a higher incidence of CVST than previously reported, ranging from 1.16 to 1.75 per 100,000 people per year based primarily on data from high-income countries (2, 3). Owing to the recent coronavirus disease 2019 (COVID-19) pandemic, the incidences of CVST has increased, and several cases of COVID-19 infection and vaccination-related CVST have also been reported (4, 5). The typical clinical manifestations include acute or subacute headache, focal neurological deficits, and seizures (6). Although anticoagulation is recommended as the preferred treatment (1, 6, 7), some patients still have a poor prognosis, with a mortality rate of 8.2%. A higher mortality rate of 38% was reported for CVST patients who experienced occurred coma (8). Moreover, patients with ICH-related CVST may have two-fold higher mortality rate than that of those without ICH (9), making anticoagulant treatment challenging. Recanalization failure or the presence of persistent thrombosis in the cerebral venous sinus may have contributed to these results. Therefore, in cases of ineffective anticoagulant therapy or disease deterioration, more aggressive treatment options such as endovascular therapy, may be considered alternative therapies (1, 6, 7, 10). However, experience with endovascular therapy (EVT) is only based on case series and not on controlled trials, and its safety and efficacy remain unclear. Thus, more evidence is needed to determine the optimal treatment choice for CVST combined with ICH in patients experiencing severe symptoms or who do not respond to anticoagulant therapy.

Despite the lack of evidence from large randomized controlled studies, EVT has been shown to achieve a proportionate rate of vascular revascularization and clinical symptom relief in a series of patients with CVST. In the TO-ACT trial, the rate of complete recanalization was higher and the rate of major hemorrhagic complications was lower in the EVT arm, suggesting that EVT may be relatively effective and safe. The reported methods of endovascular therapy include direct aspiration (11), stent retriever thrombectomy (12), balloon-assisted mechanical thrombectomy (13–15), catheter thrombolysis (16), and a combination of these methods (15, 17). Balloon dilatation not only flattens and breaks the thrombus, but also dilates the stenosis of the diseased vein and increases the surface area of the thrombi for chemical thrombolysis activity, thereby improving the efficiency of thrombus clearance and vessel restructuring (13). Moreover, we found that the sliding of the inflated balloon within the cerebral venous sinus could enhance thrombus fragmentation, which facilitated thrombus aspiration and dissolution. In endovascular thrombolysis (ET), the thrombus is dissolved by a thrombolytic substance injected into the occluded sinuses. The drug is delivered where needed and downstream from areas of vascular congestion, which helps to dissolve residual thrombus and prevent regrowth. However, there is still a lack of evidence from randomized controlled trials on balloon-assisted thrombectomy, and even relevant case reports are limited. Therefore, we retrospectively analyzed the clinical data of patients who underwent sliding balloon-assisted thrombectomy combined with aspiration and intrasinus urokinase thrombolysis at our center to investigate its safety and efficacy and to provide essential clinical evidence for the use of balloon-assisted thrombectomy for the treatment of cerebral venous sinus thrombosis.

## Methods

According to the current consensus of CVST (1) and previous evidence, we have determined the indications and timing for endovascular treatment as follows: (1) Clinical or imaging progression after the initial anticoagulation therapy; (2) Impaired consciousness; (3) Contraindication to anticoagulation therapy; (4) Imaging findings suggesting venous infarction with hemorrhagic transformation or intracerebral hemorrhage. A total of 10 patients who were diagnosed with CVST by digital subtraction arteriography (DSA) at the Department of Neurology, Jiangmen Central Hospital, between February 2022 and June 2023 met criteria and provided informed consent for endovascular treatment.

The procedure was performed under local anesthesia. Lidocaine was used for local anesthesia. For patients with mental irritability who did not cooperate, appropriate doses of dezocine or diazepam were used for sedation and analgesia, and appropriate limb restraint was used when necessary to ensure the successful completion of the operation. The conventional “Seldinger” technique was used for femoral artery puncture and cerebral angiography to determine the extent and quantity of venous sinus thrombosis. An 8F 90 cm vascular sheath (Neuron MAX, COOK Flexor Check-Flo Introducer, Cook Medical, Bloomington, IN, United States) was placed in the distal segment of the internal jugular bulb through the right femoral vein. Then, a bolus dose of heparin (2,000–3,000 U) followed by 1,000 U every hour, was intravenously administered during the procedure. A 0.018-inch microguide wire was carefully advanced through the thrombus to the distal segment beyond the microguide catheter, the tip of the microguide wire was kept in the distal segment beyond the thrombus, and the microcatheter was pulled out. An ACE60 aspiration catheter was sent to the occluded venous sinus. Vacuum negative pressure was connected to the end of the aspiration catheter, and the thrombus was aspirated repeatedly from the distance to the near. A 5-mm rapid exchange balloon guided by a 0.018 guide wire was advanced to the distal segment beyond the thrombus. Connecting with pressure pump, the balloon was gradually inflated (10–16 atm) along the thrombus segment. And the balloon was gradually inflated to the nominal pressure along the thrombus segment, and the balloon pressure was maintained. The balloon was slid repeatedly back and forth under the guidance of a microguide wire in the thrombus segment to crush the thrombus. The process was repeated three or more times until the inflated balloon could move back and forth freely. In addition, thrombus aspiration by 60-mL syringe which can produce −26 ~ 28 inches/Hg pump vacuum pressures (18) was repeated after each sliding balloon-assisted thrombectomy until no thrombus was extracted. If there was residual severe stenosis limiting blood flow, a stent could be implanted to relieve the stenosis. During the procedure, cerebral angiography was repeated to evaluate the venous outflow pathway.

After withdrawing the suction catheter and long sheath under the guidance of a 0.018 guide wire, the microcatheter was sent to the distal end of the thrombus. A bolus dose of 200,000 U of urokinase was slowly injected, and followed by continuous administration of urokinase (30,000 U/h, total 720,000 U/day) for a total of 5 days was administered into the cerebral venous sinus by microcatheter. To prevent microcatheter blockage, we used continuous microcatheter infusion of normal saline and combined with subcutaneous low molecular weight heparin (LMWH) to prevent microcatheter blockage. LMWH was continued during hospitalization, and all patients started long-term treatment with oral warfarin (maintaining an international normalized ratio between 2 and 3) or a 6-month course of rivaroxaban anticoagulation. Follow-up CTV/DSA was performed for 3 months after discharge to evaluate the imaging outcomes. Complete recanalization was defined as recanalization of all the occluded sinuses on DSA or CTV. Partial recanalization was defined as complete recanalization of one sinus but persistent occlusion of other sinuses, or partial recanalization of one or more sinuses.

This study was approved by the human Ethics Committee of Jiangmen Central Hospital (JCEC-2024-233A) and was conducted according to the principles expressed in the Declaration of Helsinki. All patients signed informed consent before enrollment.

## Results

In this retrospective observational study, a total of 10 CVST patients were ultimately enrolled from February 2022 to June 2023. The age ranged from 18 to 68 years. Males and females were equally represented. All the patients experienced headaches and gradual worsening of consciousness (10/10). Half of the patients had seizures (5/10). On neuroimaging, all patients had intracranial hemorrhage, including lobar hemorrhage, subdural hemorrhage, or subarachnoid hemorrhage (10/10), and a few of them had venous cerebral infarction (2/10). The superior sagittal sinus (SSS), transverse sinus (TS), sigmoid sinus (SiS), and jugular vein (JV) were the anatomical sites most prone to thrombosis. The common risk factors were oral contraceptives and hyperthyroidism; however, potential risk factors cannot be identified in a subset of patients (see Table 1).

**Table 1:** Demographic characteristics.

Case	Age, sex	Onset to diagnosis	GCS	mRS	Clinic manifestation				Imaging findings		DDi	Risk factor
					Epileptic seizure	Headache	Coma	Mental status	Brain lesions	Sinus involved	mg/L	
1	57, M	5d	12	5	+	+	−	Drowsiness	Infarct and ICH, left parietal lobe	SSS, Bi-TS, RSiS, RJV	7.3	Unidentified
2	61, F	30d	15	3	−	+	−	−	ICH, left temporooccipital lobe	SSS, RTS, LSC	0.82	Unidentified
3	43, F	2d	8	3	+	+	−	Lethargy	ICH, left frontoparietal lobe and the right temporooccipital lobe	SSS, SC, Bi-TS, Bi-SiS	4.18	Unidentified
4	52, M	1d	11	5	+	+	−	Cloudiness	ICH, left temporal lobe	LTS, LSiS, LJV	3.3	Unidentified
5	29, F	4d	13	5	−	+	−	Syncope	Infarct and ICH, left temporooccipital lobe	LTS, LSiS, LJV	7.35	Pregnancy
6	46, F	4d	11	5	+	+	−	Retarded, delirium	ICH, left temporooccipital	LTS, LSiS, LJV	0.84	Oral contraceptives, hyperthyroidism
7	35, F	5d	13	5	−	+	−	Retarded	ICH, left temporooccipital lobe	SSS, SC, Bi-TS, LSiS, LJV	17.68	Oral contraceptives
8	18, M	4d	15	4	+	+	−	Retarded	ICH, left frontal lobe	SSS, SC, Bi-TS, Bi-SiS	2.67	Hyperthyroidism
9	41, M	8d	7	5	−	+	−	Cloudiness	SAH	SSS, RTS, RSiS, RJV	2.07	Ruptured cerebral aneurysm
10	44, M	3d	15	4	−	+	−	Lethargy	ICH, bilateral occipital lobes, right corpus callosum and frontal lobe	RTS, RSiS, RJV	0.55	Hyperthyroidism, bacteremia

GCS, Glasgow scale; mRS, modified Rankin score; DDi, D-dimer; ICH, Intracraninal hemorrhage; SSS, Superior sagittal sinus; TS, Bilateral transverse sinus; SiS, Sigmoid sinus; JV, Internal jugular vein; SC, Sinus confluence; SAH, Subarachnoid hemorrhage.

**Figure 1:**
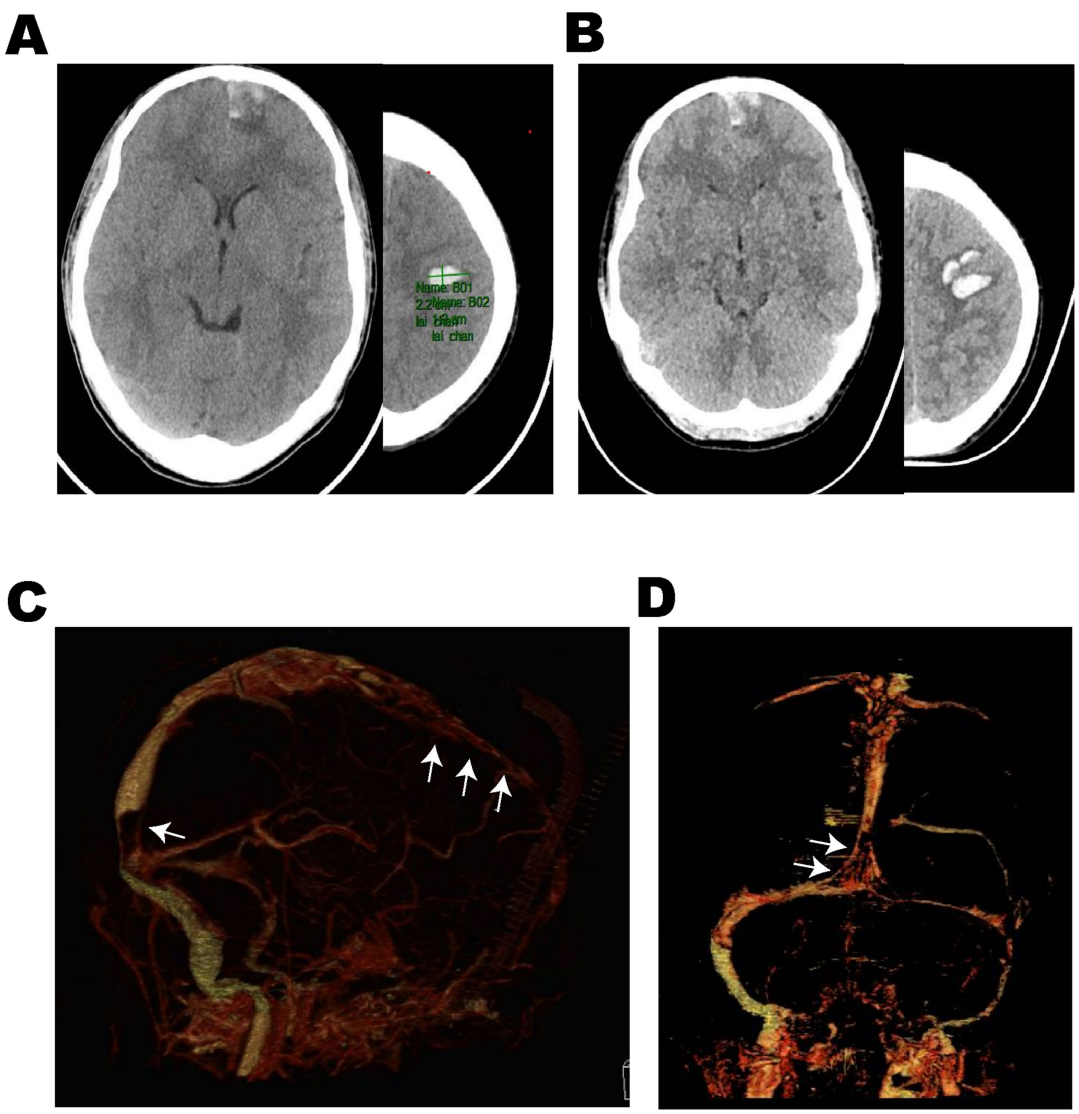
Case 8: An 18-year-old male adolescent was admitted with a 1-day history of sudden headache and limb convulsions. **(A)** Emergency head non-contrast CT scan (2023-May 28) showed multiple hematomas in the left frontal lobe. After low molecular weight anticoagulant therapy, dehydration and antiepileptic treatment, the mental state became worse, headache and limb convulsions could not be controlled. **(B)** The reexamination of head non-contrast CT (2023-May 30) showed the cerebral hemorrhage progressed. **(C,D)** Cranial CTV (2023-May 30) suggested multiple filling defects (White arrows) in the superior sagittal sinus and sinus confluence and the possibility of cerebral venous sinus thrombosis was considered. CTV computed tomography venography.

**Figure 2:**
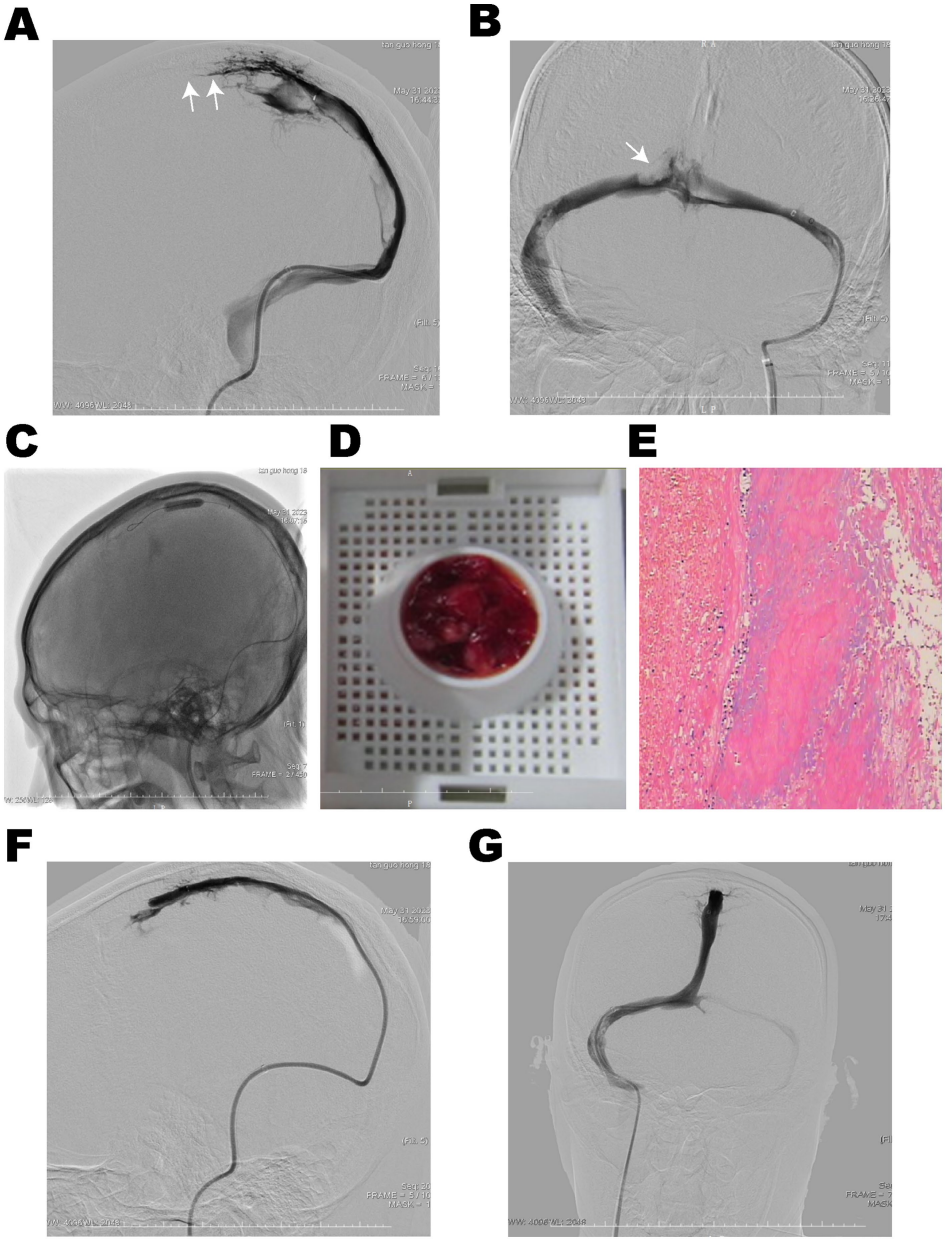
DSA showed thrombosis in the superior sagittal sinus, sinus confluence, bilateral transverse sinus, and sigmoid sinus. **(A,B)** The lumen of the superior sagittal sinus and sinus confluence was occluded (White arrows). **(C)** The balloon maintained nominal pressure along the thrombus segment for sliding thrombectomy in the superior sagittal sinus. **(D)** Combined with intermittent aspiration, a large amount of red thrombus was extracted, and **(E)** pathological examination revealed mixed type of thrombus. Cerebral venous sinus angiography showed recanalization of the superior sagittal sinus **(F)** and sinus confluence **(G)**.

**Figure 3:**
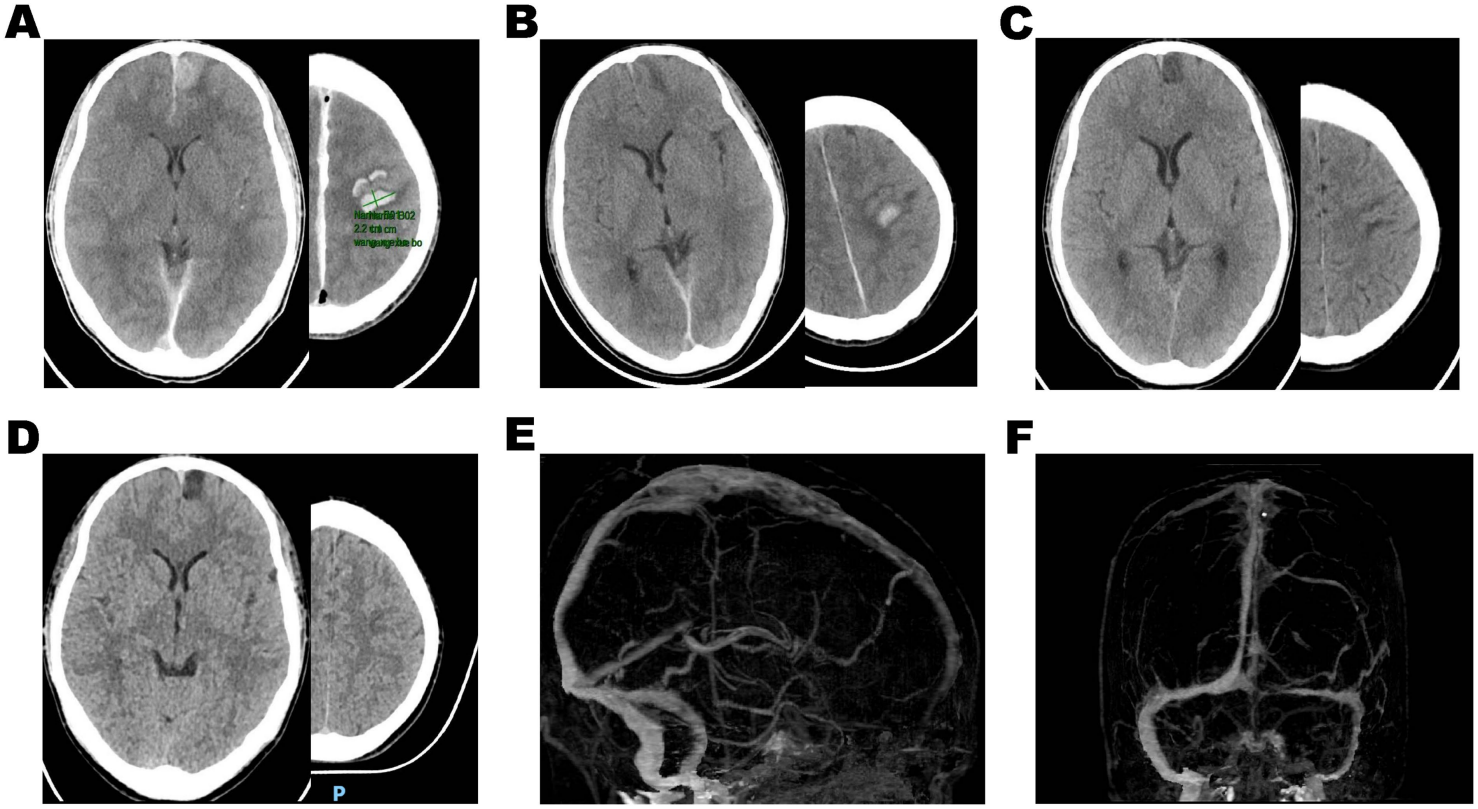
**(A)** Postoperative head non-contrasted CT demonstrated multiple bleeding in the left frontal lobe, and the volume of hematoma did not enlarge. **(B–D)** The cerebral hematoma gradually absorbed after continuous urokinase direct thrombolysis in the venous sinus following the thrombectomy. Three months after discharge, cranial CTV showed complete recanalization of the superior sagittal sinus **(E)** and sinus confluence **(F)**.

Of the 10 patients, only 5 received anticoagulant therapy before the intervention. One patient was treated with burr-hole drainage due to massive intracranial hematoma, another patient was treated with embolization due to ruptured left middle cerebral artery aneurysm. Anticoagulation was contraindicated before interventional therapy in both cases. The other 3 patients underwent emergency endovascular recanalization due to mental disaorder, without anticoagulant therapy before operation. All 10 patients underwent endovascular treatment with balloon-assisted thrombectomy and aspiration, and underwent intrasinus urokinase thrombolysis after the intervention. Residual stenosis in the cerebral venous sinus was found in 4 patients after balloon thrombectomy and intravascular aspiration, and these patients underwent stent implantation for better reperfusion. The mean operative duration was 187.8 min. Nine patients were received anticoagulant therapy after endovascular treatment, and 1 patient was treated with intravenous tirofiban for 3 days after stent implantation, followed by aspirin and clopidogrel for 3 months. Complete recanalization of the cerebral venous sinus was achieved in 7 patients, and partial recanalization was confirmed in 3 patients by postoperative DSA. During the follow-up period, cerebrovascular imaging (cranial CTV, MRV, or DSA) revealed thrombosis regrowth in 3 of the 7 patients who achieved complete recanalization, while partial recanalization of the cerebral venous sinus remained. Complete recanalization was achieved in 2 of 4 patients who achieved partial recanalization after anticoagulation. The symptoms of 10 patients were significantly relieved after the intervention. Neurological independence was confirmed at 3-and 6-month follow-up after the operation. None of these patients experienced adverse effects, such as increased cerebral hemorrhage, pulmonary embolism and neurological symptoms after the operation. These results are showed in Table 2 and Figures 1–3 illustrate the diagnosis and treatment of one of these patients.

**Table 2:** Therapy and prognosis.

Case	Preoperative anticoagulation	Onset to operation	Anti-seizure	Endovascular treatment			Operation duration (min)	Postoperative anticoagulation	Recanalization		mRS			Complications	
				A	B	C			Post-operation	Following-up	Discharge	3 m	6 m	CHE	PE
1	−	5d	Sodium valproate 0.5 g Bid	+	−	+	123	Enoxaparin, rivaroxaban	Complete	Complete	3	1	1	−	−
2	Enoxaparin, 11d	43d	−	+	−	+	160	Enoxaparin, warfarin	Partial	Partial	2	1	0	−	−
3	LMWH, 1d	3d	Sodium valproate 0.5 g Bid+ levetiracetam 0.25 g Bid	+	−	+	110	LMWH, rivaroxaban	Partial	Complete	4	2	0	−	−
4	Contraindication	13d	Sodium valproate 0.5 g Bid	+	+, LTS, LSiS	+	302	Rivaroxaban	Complete	Complete	3	1	1	−	−
5	−	1d	−	+	+, RSiS	+	375	Nadroparin, warfarin	Complete	Partial	0	0	0	−	−
6	LMWH, 1d	5d	Levetiracetam 0.5 g Bid	+	−	+	190	LMWH, rivaroxaban	Partial	Complete	3	1	1	−	−
7	Nadroparin, 3d	4d	−	+	−	+	120	Nadroparin, rivaroxaban	Complete	Partial	3	1	0	−	−
8	Nadroparin, 1d	4d	Levetiracetam 0.5 g Bid	+	−	+	183	Nadroparin, rivaroxaban	Complete	Complete	1	1	1	−	−
9	Contraindication	7d	−	+	+, RTS	+	165	LMWH, rivaroxaban	Complete	Complete	3	1	1	−	−
10	−	1d	−	+	+, RTS, RSiS, RJV	+	150	Intravenous tirofiban, dual antiplatelet	Complete	Partial	2	2	1	−	−

Balloon thrombectomy + aspiration.

B stent implantation.

C sequential catheter thrombolysis.

mRS, modified Rankin Score; LMWH, Low molecular weight heparin; SSS, Superior sagittal sinus; TS, Bilateral transverse sinus; SiS, Sigmoid sinus; JV, Internal jugular vein; SC, Sinus confluence; SAH, Subarachnoid hemorrhage; CHE, Cerebral hematoma enlarge; PE, Pulmonary embolism.

Case 4 was treated with burr-hole drainage due to massive intracranial hematoma, and case 9 was treated with embolization due to ruptured left middle cerebral artery aneurysm. Anticoagulation was contraindicated before EVT in both cases.

## Discussion

In our case series, sliding balloon-assisted thrombectomy combined with intrasinus urokinase thrombolysis resulted in effective recanalization without rebleeding or neurological deterioration in patients with hemorrhagic cerebral venous sinus thrombosis. The symptoms of most patients were rapidly alleviated, leading to a favorable neurological recovery during the late follow-up period. This limited evidence also indicated that sliding balloon-assisted thrombectomy was efficient and safe for the treatment of hemorrhagic cerebral venous sinus thrombosis. Sequent intrasinus urokinase thrombolysis in the venous sinus after balloon thrombectomy can promote the dissolution of the unaspirated thrombus in the venous sinus, facilitate the clearance of the thrombus, and prevent embolic events caused by the detachment of the venous thrombus. These results indicate that our approach of sliding balloon thrombectomy and aspiration followed by catheter-directed thrombolysis was safe and effective for treatment of hemorrhagic cerebral venous sinus thrombosis in patients with severe thrombus burden, thereby providing an effective treatment option for selected CVST patients.

At present, standard anticoagulant therapy is considered a safe first-line treatment according to the current guidelines (1), but its vascular recanalization rate is only 60–67% (19), which may prevent symptom relief and lead to an unfavorable prognosis for CVST patients. In a previous study involving 624 patients with CVST, the incidence of poor outcomes was 21% and the mortality rate was 8.2%, despite the use of standard anticoagulant therapy (8). Complete recanalization has been shown to be associated with improved outcomes (20). These findings suggest that cerebral venous sinus recanalization plays a key role in quickly relieving symptoms and improving patient prognosis. In addition, coma CVST patients exhibit a mortality rate of 38%, and the presence of ICH is also a poor prognostic factor that almost doubles the risk of mortality (8). Therefore, it has been proposed that more aggressive treatment should be considered, particularly for patients with severe CVST in the following four situations: (i) intracranial hematoma; (ii) coma; (iii) mental symptoms; and (iv) intracranial deep vein thrombosis (21).

There is a low level of evidence for the use of endovascular treatment as a second-line treatment for venous sinus thrombosis in current guidelines due to the lack of large randomized controlled trials (6, 10, 22). In recent years, a growing body of evidence has focused on endovascular treatment for CVST, and different degrees of recanalization have been achieved. The common methods include local thrombolysis in the venous sinus, retriever stent thrombectomy, aspiration, balloon-assisted thrombectomy, or a combination of the two methods (23).

Systemic or local thrombolysis treatment has some theoretical advantages over heparin treatment. Thrombolytic drugs can directly dissolve the thrombus so it can be removed within hours. If successful, recanalization can be more frequently and rapidly achieved than with heparin alone (24–26). Although it has been reported that systemic or local thrombolysis has an effect on venous sinus recanalization and restoring neurological independence in some patients, the risks of cerebral hemorrhage and mortality after thrombolytic therapy have also been mentioned (27, 28). Alteplase (atpa) and urokinase are the most commonly used drugs for local thrombolysis. A systematic review reported that about 90% of patients with atpa who underwent local thrombolysis achieved varying degrees of cerebral venous sinus recanalization, and the overall incidence of cerebral hemorrhage was only 2.94% (16). In another report, 11 severe CVST patients during puerperium were treated with local urokinase thrombolysis and heparin anticoagulation, of which 9 patients achieved complete recanalization, 2 patients achieved partial recanalization, and basically got a good prognosis (29). In addition, other investigators also reported the combined use of devices and procedures such as aspiration thrombectomy, Fogarty catheters, balloon angioplasty, and the AngioJet system to fragment or remove clots before intrasinus local thrombolysis (23, 30–32). And most part of CVST patients also could achieve a good prognosis. These clues suggested that local thrombolysis may be a safe and effective treatment. However, large-sample randomized controlled trials to determine the efficacy and safety of systemic or local thrombolysis are lacking, and there is a lack of evidence on the type, dosage, and duration of thrombolytic agents to determine the optimal local thrombolysis.

Although stent thrombectomy is commonly used in cerebral artery occlusion diseases, it seems to be less applied alone in the treatment of CVST patients. It may be due to the smaller diameter of the thrombectomy stent than the venous sinus [the maximum diameter of commonly used stents is mostly 6 mm (33)], insufficient deployment force, and the low efficiency of thrombus capture caused by the characteristics of the stent structure (23). In addition, the complexity of the process of repeated opening and retrieval also affects the use of this method. In a meta-analysis, 55 patients with CVST who still had progression after anticoagulation were retrospectively included. For patients treated with stent retrievers and catheter aspiration, only 36% achieved complete recanalization and 72% had a favorable clinical outcome (34).

AngioJet rheolytic catheter and Penumbra system are the most commonly used thrombectomy techniques in the literature. As AngioJet is a bulky and rigid device, complete recanalization was achieved in only 55% of patients treated with AngioJet (23). Compared with AngioJet, the Penumbra system has a smaller size, which allows massive thrombus removal by aspiration without a need for completely remove the catheter. Choulakian and Alexander (35) reported the first use of Penumbra system for lysing CVST in 4 patients and Siddiqui et al. (36) reported the use of Penumbra 0.54, which has a bigger lumen that allows compatibility with other microcatheters in treated 2 patients. Good recanalization was obtained in all reported cases. However, these two aspiration techniques have equipment reliance, which affects their application.

In the current evidence, balloons are often used in combination with aspiration thrombectomy to treat patients with CVST. Matsuda et al. (37) reported the “venous sinus floss” technique for recanalization of acute sinus thrombosis. Three patients with venous sinus thrombosis were treated with balloon dilatation combined with aspiration technique (AC64). The recanalization effect was satisfactory. Shui et al. (13) reported a case series treated with balloon dilation and thrombectomy. All 26 patients achieved cerebral venous sinus recanalization and achieved good prognosis. However, these clues have obvious limitations, including reporting bias due to small sample sizes and inconsistent inclusion criteria. The low incidence of CVST makes it difficult to carry out large-scale clinical studies, so more research data are needed to explore better treatment options.

In this study, the use of balloon assisted thrombectomy combined with aspiration and subsequent intrasinus urokinase thrombolysis in patients with hemorrhagic venous sinus thrombosis improved the recanalization rate (10 patients showed partial or complete recanalization after treatment), and efficiently and rapidly relieved clinical symptoms so that patients could obtain independent neurological function. The results may be explained by the following reasons: on the one hand, early recanalization alleviates intracranial hypertension and cerebral blood circulation disorder, which promotes the absorption of hematoma and rapid relief of symptoms. On the other hand, even if venous sinus thrombosis recurred in some patients, the recanalization provided enough time to form more cerebral venous collateral branches to compensate for the defect of the main trunk occlusion, so no new symptoms occurred. There are some advantages of sliding balloon thrombectomy combined with aspiration over previous endovascular treatment as follows: (1) Enhancing the crushing effect on large pieces of thrombi due to the inflated balloon with stable inflation pressure sliding along the microguide wire, resulted in a more significant impact on thrombus fragmentation and increasing the surface area of the thrombus exposed to thrombolytics. (2) Increasing thrombus clearance efficiency as a result of intermittent aspiration of thrombus fragments in the cerebral venous sinus after balloon thrombectomy. (3) Severe pulmonary embolism prevention as a result of the inability of fragmented thrombi to return to the pulmonary artery due to continuous infusion of urokinase directly into the venous sinus facilitating the dissolution of the remaining thrombus that cannot be fragmented or aspirated. (4) The technique does not rely on complicated suction system, and the operation is relatively simple, which makes the technique easier to promote.

However, there were several limitations in the study. First, this was an observational study with a small sample of patients from a single center who could not be compared with patients receiving other treatments by statistical methods, and the advantages and disadvantages of this approach compared with those of other treatments remain unknown. Second, the clinical symptoms of the enrolled patients seemed to be mild, and there was a lack of patients with severe neurological symptoms, which affected the ability to effectively observe patients with severe clinical manifestations. Third, we did not monitor the activated clotting time (ACT) before and after heparinization to guide the perioperative heparin dosage. Finally, there is no consensus regarding the optimal method for identifying suitable patients for combined mechanical thrombectomy to maximize the benefits of this therapy. Therefore, large-sample randomized controlled trials are urgently needed in the future to determine the efficacy and safety of this hybrid endovascular treatment in patients with hemorrhagic CVST.

## Conclusion

In this case series, we report that the combination of sliding balloon thrombectomy with aspiration and intrasinus urokinase thrombolysis is safe and effective in the treatment of patients with hemorrhagic cerebral venous sinus thrombosis. Early and appropriate endovascular treatment may effectively alleviate the suffering of patients and improve disease prognosis. A randomized controlled trial is needed to confirm these findings to provide effective and safe treatment options for the treatment of CVST.

## Data Availability

The datasets presented in this article are not readily available because of ethical and privacy restrictions. Requests to access the datasets should be directed to the corresponding authors.
